# Supplementary material for: miR-215 Targeting Novel Genes EREG, NIPAL1 and PTPRU Regulates the Resistance to E.coli F18 in Piglets
Source: Genes (Basel). 2020 Sep 7;11(9):1053. doi: 10.3390/genes11091053 (PMC7563519; doi:10.3390/genes11091053)
Supplement: Supplementary file 1 [file genes-11-01053-s001.zip › genes-889956-supplementary/Supplementary Materials.doc]

miR-215 targeting Novel Genes *EREG*, *NIPAL*1 and *PTPRU* Regulates the Resistance to *E.coli* F18 in Piglets

Chao-Hui Dai ^1,a^, Fang Wang ^1,a^, Shi-Qin Wang ^1^, Zheng-Chang Wu ^1^, Sheng-Long Wu ^1,2^ and Wen-Bin Bao ^1,2,^*

^1^	Key Laboratory for Animal Genetics, Breeding, Reproduction and Molecular Design of Jiangsu Province, College of Animal Science and Technology, Yangzhou University, Yangzhou 225009, P. R. China; dx120170081@yzu.edu.cn (C.D.); w17351373585@163.com (S.W.); wangfangRD@163.com (F.W.); zcwu@yzu.edu.cn (Z.W.)

^2^	Joint International Research Laboratory of Agriculture & Agri-Product Safety, Yangzhou University, Yangzhou, Jiangsu, P. R. China; slwu@yzu.edu.cn (S.W.)

*****	Correspondence: wbbao@yzu.edu.cn, Tel. : +86-514-87979316

^a^	These authors contributed equally to this study.

**Figure S1.** Sequencing results of firefly luciferase recombinant vectors for miR-215 target genes. The sequences marked with a red box represent the recombination sequences.

**Figure S2.** Sequencing results of miR-215 target genes interference vectors. The sequences marked with a red box represent the recombination sequences.

**Figure S3.** ELISA standard curves of pro-inflammatory cytokines TNF-α and IL-1β.

**Figure S4.** Sequencing results of wild-type and mutant luciferase vectors in the core promoter region of miR-215 target genes. The sequences marked with a red box represent the recombination sequences.

**Table S1.** Information of quantitative PCR primers

| Name | Sequence of the primer | Length of products (bp) | Accession number |
| --- | --- | --- | --- |
| *IDO1* | F: 5'-CATCACCATGGCGTACGTGT-3'  R: 5'-TTTGCCAAGACACAATCTGCG-3' | 144 | NM_001246240.1 |
| *ANK2* | F: 5'-ACGCACATCATCAACGTCCT-3'  R: 5'-CACGGAGATGTAGCCCAGAC-3' | 108 | XM_021100703.1 |
| *HSPA4L* | F: 5'-TGGGTGGCAGGAACTTTGAT-3'  R: 5'-AACGTAACAAGGCCCGAGAG-3' | 158 | XM_021101609.1 |
| *PVRL1* | F: 5'-CAACTACCACATGGACCGCT-3'  R: 5'-GCTTCACATCCATTCGCTGC-3' | 116 | NM_001001262.1 |
| *EREG* | F: 5'-TGTGACGCTGTGGCAGTTT-3'  R: 5'-CACCAGCTTGAACTCACCGC-3' | 153 | XM_013978775.2 |
| *CBL* | F: 5'-CCGGCAATGTGAAAAAGAGC-3'  R: 5'-AACCGCACCACCTTGTCC-3' | 199 | XM_003129932.4 |
| *NIPAL1* | F: 5'-TGATCTGTCCAAATGCCTCCC-3'  R: 5'-GCTCAGGTTGGTTATGCTGGAA-3' | 110 | XM_003360909.4 |
| *GPR126* | F: 5'-TTCCTTCAGGGCGAATGTGG-3'  R: 5'-TCGTTAGGGTAGCACGGAGA-3' | 172 | [XM_021087195.1](https://www.ncbi.nlm.nih.gov/nuccore/XM_021087195.1) |
| *MFAP3* | F: 5'-GCTTCAACCAAATGACCCCAC-3'  R: 5'-GACATCATCACCTGAGTGGGAA-3' | 104 | XM_003134126.4 |
| *PTPRU* | F: 5'-TCTGCAAGGCTGAGTGACAG-3'  R: 5'-AACTCTAGGGGTGGTGAGCA-3' | 200 | XM_021095731.1 |

**Table S3.** Oligo sequences for RNAi

| Name | Oligo sequences (5'→3') |
| --- | --- |
| pGMLV-EREG-F | *gatcc*gcaaagagtatgtggctttgaTTCAAGAGAtcaaagccacatactctttgcTTTTTTG |
| pGMLV-EREG-R | *aatt*CAAAAAAgcaaagagtatgtggctttgaTCTCTTGAAtcaaagccacatactctttgcG |
| pGMLV-NIPAL-F | *gatcc*ggtcaaccgtgatggttatccTTCAAGAGAggataaccatcacggttgaccTTTTTTG |
| pGMLV-NIPAL-R | *aatt*CAAAAAAggtcaaccgtgatggttatccTCTCTTGAAggataaccatcacggttgaccG |
| pGMLV-PTPRU-F | *gatcc*ggaagtcgcttctctgcatgaTTCAAGAGAtcatgcagagaagcgacttccTTTTTTG |
| pGMLV-PTPRU-R | *aatt*CAAAAAAggaagtcgcttctctgcatgaTCTCTTGAAtcatgcagagaagcgacttccG |

Note: Italicized lowercase letters represent restriction sites. The rest of lower-case letters are interference sequences and their complementary sequences. Capital letters are the linker sequence. The underlined letters represent the shRNA sequences.

**Table S4. Amplification primers for promoter region of miRNA-215 target genes**

| Name | Sequence of the primer | Length of products (bp) |
| --- | --- | --- |
| EREG-P1 | F: 5'-GG*ACTAGT*GGTGGGGGTGGGGTTCTGCTTC-3'  R: 5'-CATG*CCATGG*CCCGTCAGGGAAGTGACCT-3' | 2022 |
| EREG-P2 | F: 5'-GG*ACTAGT*GCCAGAGTCATCTTTCCTGGT-3'  R: 5'-CATG*CCATGG*CCCGTCAGGGAAGTGACCT-3' | 1791 |
| EREG-P3 | F: 5'-GG*ACTAGT*TACTAGGCACTGGGGAAAGCA-3'  R: 5'-CATG*CCATGG*CCCGTCAGGGAAGTGACCT-3' | 1555 |
| EREG-P4 | F: 5'-GG*ACTAGT*CGACATGCATTAGATTCCATTTTCC-3'  R: 5'-CATG*CCATGG*CCCGTCAGGGAAGTGACCT-3' | 1252 |
| EREG-P5 | F: 5'-GG*ACTAGT*AGCTGGAAGAGGCCAGAATG-3'  R: 5'-CATG*CCATGG*CCCGTCAGGGAAGTGACCT-3' | 1093 |
| EREG-P6 | F: 5'-GG*ACTAGT*ACTGTTGGGTCCACCTTTGT-3'  R: 5'-CATG*CCATGG*CCCGTCAGGGAAGTGACCT-3' | 799 |
| EREG-P7 | F: 5'-GG*ACTAGT*GCCATAAATGCCTGTACCCCA-3'  R: 5'-CATG*CCATGG*CCCGTCAGGGAAGTGACCT-3' | 604 |
| EREG-P8 | F: 5'-GG*ACTAGT*AGGCGGGAAGACCATCTGATT-3'  R: 5'-CATG*CCATGG*CCCGTCAGGGAAGTGACCT-3' | 374 |
| EREG-P9 | F: 5'-GG*ACTAGT*CATCCGTCAGTCCATGCTCC-3'  R: 5'-CATG*CCATGG*CCCGTCAGGGAAGTGACCT-3' | 164 |
| NIPAL1-P1 | F: 5'-GG*ACTAGT*TTTTAGGGTTGAACCGGCAG-3'  R: 5'-CATG*CCATGG*GCTCACTCCTCACCCACC-3' | 2082 |
| NIPAL1-P2 | F: 5'-GG*ACTAGT*TGCACGGCATATGGAGGTA-3'  R: 5'-CATG*CCATGG*GCTCACTCCTCACCCACC-3' | 1750 |
| NIPAL1-P3 | F: 5'-GG*ACTAGT*ACTTATGTGCAATATACGTGTCAGT-3'  R: 5'-CATG*CCATGG*GCTCACTCCTCACCCACC-3' | 1376 |
| NIPAL1-P4 | F: 5'-GG*ACTAGT*TGAGGCTTCTGCTATGAAACTCC-3'  R: 5'-CATG*CCATGG*GCTCACTCCTCACCCACC-3' | 1088 |
| NIPAL1-P5 | F: 5'-GG*ACTAGT*GTCCCACAAGGACCCGCTAC-3'  R: 5'-CATG*CCATGG*GCTCACTCCTCACCCACC-3' | 910 |
| NIPAL1-P6 | F: 5'-GG*ACTAGT*TGGGAATGAGTCGTTTCTGAGC-3'  R: 5'-CATG*CCATGG*GCTCACTCCTCACCCACC-3' | 706 |
| NIPAL1-P7 | F: 5'-GG*ACTAGT*GTGGGCACAACTTCCCTACA-3'  R: 5'-CATG*CCATGG*GCTCACTCCTCACCCACC-3' | 478 |
| NIPAL1-P8 | F: 5'-GG*ACTAGT*CTCCAGCACAGAATTTACGCC-3'  R: 5'-CATG*CCATGG*GCTCACTCCTCACCCACC-3' | 383 |
| NIPAL1-P9 | F: 5'-GG*ACTAGT*CCCTGTGAGGTGTGTCTGTG-3'  R: 5'-CATG*CCATGG*GCTCACTCCTCACCCACC-3' | 257 |
| PTPRU-P1 | F: 5'-CCC*AAGCTT*TTGTACAAGGGCTTACACTGC-3'  R: 5'-CATG*CCATGG*CTCGACCGTGCGTTCCTC-3' | 1991 |
| PTPRU-P2 | F: 5'-CCC*AAGCTT*TCCTTAACCCACTGCATCATAGTG-3'  R: 5'-CATG*CCATGG*CTCGACCGTGCGTTCCTC-3' | 1779 |
| PTPRU-P3 | F: 5'-CCC*AAGCTT*ACAAAGGAAACTCCAGGAAGGA-3'  R: 5'-CATG*CCATGG*CTCGACCGTGCGTTCCTC-3' | 1551 |
| PTPRU-P4 | F: 5'-CCC*AAGCTT*TGATTGCCCTCAGACCACAA-3'  R: 5'-CATG*CCATGG*CTCGACCGTGCGTTCCTC-3' | 1263 |
| PTPRU-P5 | F: 5'-CCC*AAGCTT*GTGTGGGATGCAGTGAAAGC-3'  R: 5'-CATG*CCATGG*CTCGACCGTGCGTTCCTC-3' | 1067 |
| PTPRU-P6 | F: 5'-CCC*AAGCTT*GGAGTTCCTGTTGTGGCTCA-3'  R: 5'-CATG*CCATGG*CTCGACCGTGCGTTCCTC-3' | 844 |
| PTPRU-P7 | F: 5'-CCC*AAGCTT*GTTTCCAAAGACCCCACCAC-3'  R: 5'-CATG*CCATGG*CTCGACCGTGCGTTCCTC-3' | 405 |
| PTPRU-P8 | F: 5'-CCC*AAGCTT*TGTTTGAGAGCTAGGAGTGGG-3'  R: 5'-CATG*CCATGG*CTCGACCGTGCGTTCCTC-3' | 249 |
| PTPRU-P9 | F: 5'-CCC*AAGCTT*GTGTGTGGTCCTGGAGTGG-3'  R: 5'-CATG*CCATGG*CTCGACCGTGCGTTCCTC-3' | 122 |

Note: P1-P9 represent amplification primers for different segments of gene promoter regions, respectively. The italicized letters represent the restriction sites for enzymes *SpeI*, *HindIII* and *NcoI*, respectively.

**Table S5.** BSP Primers Information

| Name | Sequence of the primer | Length of products (bp) | Annealing temperature |
| --- | --- | --- | --- |
| NIPAL1-BSP | F: 5'-GAAGGGGGTTTTAGTATAGAATTTA-3'  R: 5'-ACACAAACACACCTCACAAAATTAA-3' | 105 | 58.1 ℃ |
| PTPRU-BSP | F: 5'-GTTAAATTGGAGTTGTAGTTGTTGG-3'  R: 5'-AATAAATTCAACTAATTCTCCTTAC-3' | 197 | 68.1 ℃ |

**Table S6. Wild-type and mutant oligo sequences in the core promoter region of genes**

| Name | Sequence of the oligo (5'→3') |
| --- | --- |
| pGL3-Basic-EREG-wt-F | CTGGCTCCAGTAAAAGGCAGCCATTTCCCCAAATGGGACTCCAGCATCA |
| pGL3-Basic-EREG-wt-R | *AGCT*TGATGCTGGAGTCCCATTTGGGGAAATGGCTGCCTTTTACTGGAGCCAG*GTAC* |
| pGL3-Basic-EREG-mut-F | CTGGCTCCAGTAAAGGGCAGCCATTTCCCCAAATGGGACTCCAGCATCA |
| pGL3-Basic-EREG-mut-R | *AGCT*TGATGCTGGAGTCCCATTTGGGGAAATGGCTGCCCTTTACTGGAGCCAG*GTAC* |
| pGL3-Basic-NIPAL1-wt-F | CCCTGTGAGGTGTGTCTGTGTTTTCGCACTGCTTGGTTCCCGCAGGGGTCCCGCCCTACGGACAGCAAA |
| pGL3-Basic-NIPAL1-wt-R | *AGCT*TTTGCTGTCCGTAGGGCGGGACCCCTGCGGGAACCAAGCAGTGCGAAAACACAGACACACCTCACAGGG*GTAC* |
| pGL3-Basic-NIPAL1-mut-1-F | CTCTGTGAGGTGTGTCTGTGTTTTCGCACTGCTTGGTTCCCGCAGGGGTCCCGCCCTACGGACAGCAAA |
| pGL3-Basic-NIPAL1-mut-1-R | *AGCT*TTTGCTGTCCGTAGGGCGGGACCCCTGCGGGAACCAAGCAGTGCGAAAACACAGACACACCTCACAGAG*GTAC* |
| pGL3-Basic-NIPAL1-mut-2-F | CCCTGTGAGGTTTGTCTGTGTTTTCGCACTGCTTGGTTCCCGCAGGGGTCCCGCCCTACGGACAGCAAA |
| pGL3-Basic-NIPAL1-mut-2-R | *AGCT*TTTGCTGTCCGTAGGGCGGGACCCCTGCGGGAACCAAGCAGTGCGAAAACACAGACAAACCTCACAGGG*GTAC* |
| pGL3-Basic-NIPAL1-mut-3-F | CCCTGTGAGGTGTGACTGTGTTTTCGCACTGCTTGGTTCCCGCAGGGGTCCCGCCCTACGGACAGCAAA |
| pGL3-Basic-NIPAL1-mut-3-R | *AGCT*TTTGCTGTCCGTAGGGCGGGACCCCTGCGGGAACCAAGCAGTGCGAAAACACAGTCACACCTCACAGGG*GTAC* |
| pGL3-Basic-NIPAL1-mut-4-F | CCCTGTGAGGTGTGTCTGTGTTTTCGCACTGCTTGGTTTCCGCAGGGGTCCCGCCCTACGGACAGCAAA |
| pGL3-Basic-NIPAL1-mut-4-R | *AGCT*TTTGCTGTCCGTAGGGCGGGACCCCTGCGGAAACCAAGCAGTGCGAAAACACAGACACACCTCACAGGG*GTAC* |
| pGL3-Basic-NIPAL1-mut-5-F | CCCTGTGAGGTGTGTCTGTGTTTTCGCACTGCTTGGTTCCCGCAGGGGTCCAGCCCTACGGACAGCAAA |
| pGL3-Basic-NIPAL1-mut-5-R | *AGCT*TTTGCTGTCCGTAGGGCTGGACCCCTGCGGGAACCAAGCAGTGCGAAAACACAGACACACCTCACAGGG*GTAC* |
| pGL3-Basic-NIPAL1-mut-6-F | CCCTGTGAGGTGTGTCTGTGTTTTCGCACTGCTTGGTTCCCGCAGGGGTCCCTCCCTACGGACAGCAAA |
| pGL3-Basic-NIPAL1-mut-6-R | *AGCT*TTTGCTGTCCGTAGGGAGGGACCCCTGCGGGAACCAAGCAGTGCGAAAACACAGACACACCTCACAGGG*GTAC* |
| pGL3-Basic-NIPAL1-mut-7-F | CCCTGTGAGGTGTGTCTGTGTTTTCGCACTGCTTGGTTCCCGCAGGGGTCCCGCCCTACCGACAGCAAA |
| pGL3-Basic-NIPAL1-mut-7-R | *AGCT*TTTGCTGTCGGTAGGGCGGGACCCCTGCGGGAACCAAGCAGTGCGAAAACACAGACACACCTCACAGGG*GTAC* |
| pGL3-Basic-PTPRU1-wt-F | CATTGAGAGGATGAAAAGGAATACTGTGAACAACTCTAAAAGCTAGAGTTTCCAAAGACCCCACCAC |
| pGL3-Basic-PTPRU1-wt-R | *AGCT*GTGGTGGGGTCTTTGGAAACTCTAGCTTTTAGAGTTGTTCACAGTATTCCTTTTCATCCTCTCAATG*GTAC* |
| pGL3-Basic-PTPRU1-insert-F | CATTTGAGAGGATGAAAAGGAATACTGTGAACAACTCTAAAAGCTAGAGTTTCCAAAGACCCCACCAC |
| pGL3-Basic-PTPRU1-insert-R | *AGCT*GTGGTGGGGTCTTTGGAAACTCTAGCTTTTAGAGTTGTTCACAGTATTCCTTTTCATCCTCTCAAATG*GTAC* |
| pGL3-Basic-PTPRU1-mut-F | CATTGAGAGGATGAAAAGGAATTCTGTGAACAACTCTAAAAGCTAGAGTTTCCAAAGACCCCACCAC |
| pGL3-Basic-PTPRU1-mut-R | *AGCT*GTGGTGGGGTCTTTGGAAACTCTAGCTTTTAGAGTTGTTCACAGAATTCCTTTTCATCCTCTCAATG*GTAC* |

Note: wt represents wild type, mut represents point mutation; insert stands for insertion mutation. The italicized letters represent restriction sites for enzymes *KpnI* and *HindIII*, respectively.
